# BrinjalFruitX: A field-collected image dataset for machine learning and deep learning-based disease identification in brinjal fruits

**DOI:** 10.1016/j.dib.2026.112490

**Published:** 2026-01-21

**Authors:** Abu Kowshir Bitto, Md. Zahid Hasan, Md. Hasan Imam Bijoy, Khalid Been Badruzzaman Biplob, Mohammad Mahadi Hassan, Mohammad Shohel Rana, Abdul Kadar Muhammad Masum

**Affiliations:** a Department of Software Engineering, Daffodil International University, Dhaka 1216, Bangladesh; b Department of Computer Science and Engineering, Daffodil International University, Dhaka 1216, Bangladesh; c Department of Computer Science and Engineering, International Islamic University Chittagong, Chattogram 4318, Bangladesh; d Department of Computer Science and Engineering, Southeast University, Dhaka 1208, Bangladesh

**Keywords:** Eggplant dataset, Disease classification, Agriculture informatics, Deep learning, Computer vision

## Abstract

Brinjal (Solanum melongena) or eggplant is one of the four most essential vegetable crops that are grown in Bangladesh and contribute significantly to the agricultural industry of the country. Brinjal supports the livelihood of numerous small farmers; however, brinjal is severely susceptible to various fruit diseases, which have serious impacts on yield quality and may cause considerable economic losses. While most existing plant disease datasets primarily focus on leaf-related disorders, only a limited number include fruit-related diseases and even those contain very few classes. This gap is significant because fruit diseases directly affect crop quality, market value, and overall yield. This is why we present here a new and comprehensive dataset that is unparalleled, exclusively for brinjal fruit diseases. This data set consists of 1823 high-quality, labelled images, across five distinct classes: Phomopsis Blight, Shoot and Fruit Borer, Fruit Cracking, Wet Rot, and Healthy Fruit. The images were collected from real farm conditions in numerous areas of Bangladesh to ensure a robust sample of varied environmental and farming practices impacting the growth of diseases. This dataset is designed with the unique aim to support plant disease research and enhance training of deep learning models for autonomous disease detection. Lastly, the dataset will allow early disease detection, enhancing crop management practice, reduction of losses, and increasing farmers' economic returns. The release of this dataset will encourage agricultural research as well as practical use in precision agriculture.

Specifications Table

**Table:** 

Subject	Computer Science
Specific subject area	Image Classification, Image Identification, Deep Learning, and Computer Vision
Type of data	Image (.JPG)
Data collection	The data was collected from various farming areas in Bangladesh, including Bogura, and Dhaka, during January and February 2025. It represents a wide range of environmental conditions, captured with high-resolution mobile cameras like the Poco F3 and Samsung A52. The aim of the dataset is to support deep learning applications for disease detection, ultimately helping farmers manage crops more effectively and reduce financial losses caused by fruit diseases.
Data source location	Bogura, Bangladesh Latitude: 24.8483° N, Longitude: 89.2220° E Zone: Rajshahi, Country: Bangladesh Dhaka, Bangladesh Latitude: 23.8103° N, Longitude: 90.3200° E Zone: Dhaka, Country: Bangladesh
Data accessibility	Repository name: Mendeley Data… Data identification number: 10.17632/ngc58fsxgd.1 Direct URL to data: https://data.mendeley.com/datasets/ngc58fsxgd/1
Related research article	None

## 1 Value of the Data

• This dataset gives a pioneering and highly specific collection of images committed to brinjal (eggplant) fruit disease in isolation, an area significantly underrepresented within existing plant disease collections. It consists of 1823 high-resolution images organized into five distinct classes: Phomopsis Blight, Shoot and Fruit Borer, Brinjal Fruit Cracking, Wet Rot, and Healthy Fruit. This dataset is crucial to the search for early disease identification in brinjal, a crucial crop in Bangladesh, as well as the closing of an important knowledge gap in plant disease research.
• The images contained in this dataset were captured under actual-field conditions under different weather conditions, and thus it is extremely suitable for training deep learning models for real-field, ground-level disease identification. This renders it a fundamental instrument in the development of AI-powered tools for brinjal crop health management by farmers to prevent economic loss due to fruit diseases. With the incorporation of photographs captured under varied environmental conditions, this dataset ensures the promise of creating consistent models that can perform better and working under changing agricultural conditions.
• Unlike most datasets available to date, focusing primarily on leaf diseases, only a limited number include fruit-related diseases and even those contain very few classes. This dataset directly targets fruit-specific diseases, marking a pioneering work in plant disease research. Its unique feature of focusing on diseases of brinjal fruits will help researchers develop customized algorithms capable of detecting issues at the level of the fruit because of diseases, thus improving the quality and marketability of brinjal. This holds a tremendous scope for agricultural growth and improved measures for controlling diseases for offering better-quality produce and greater revenue for farmers.
• This rich image dataset of diverse images is well-suited to a range of machine learning applications, including image classification, disease diagnosis, and crop management optimization. Its heavy labeling of disease classes offers a solid platform upon which to build diagnostic models that can be immediately employed in precision farming systems to allow farmers to make timely, informed decisions about pesticide application, harvesting, and post-harvest treatment.
• The dataset’s design also makes it suitable for use in the development of Internet of Things (IoT) systems and smart agricultural technologies. Adding this dataset to real-time monitoring software helps researchers develop smart sensors that can detect disease at the onset, providing farmers immediate alert. This integration can enhance food traceability, ensure better food safety standards, and reduce the application of chemical treatments, thus facilitating sustainable agriculture.
• Apart from its direct use in detecting brinjal disease, this dataset has broader applications in agricultural technology, such as crop disease monitoring and public health surveillance. It is beneficial for domains like computer vision, agricultural informatics, and decision-making systems based on AI. Such a rich dataset can enable researchers to benchmark algorithms, compare the performance of disease detection systems, and further research for automated quality control systems for agriculture.

## 2 Background

The main motivation behind preparing this dataset is to aid in solving the serious issue of brinjal (eggplant) plant disease detection, a vital crop of Bangladesh. Brinjal falls among the top four most valuable vegetable crops of Bangladesh, and many small-scale farmers' livelihood relies on it. However, brinjal is highly susceptible to various fruit diseases, which have a negative effect on yield and price. Although fruit-related diseases are important, there was a major gap in all the available data sets focusing primarily on leaf diseases and not fruit-related diseases. Only a limited number include fruit-related diseases and even those contain very few classes and an insufficient number of samples. It was with the aim to bridge this gap and assist in the development of machine learning and computer vision models for the detection of brinjal fruit disease at an early stage that this data set was established.

The data set was created by taking high-resolution pictures of brinjal fruits with five various diseases—Phomopsis Blight, Shoot and Fruit Borer, Brinjal Fruit Cracking, Wet Rot, and Healthy Fruit—straight from real farm conditions from across Bangladesh. The pictures were taken from various farming regions, like Bogura, Dhaka, to ensure variety in environmental conditions such as lighting, weather, and humidity. This approach ensures that the dataset reflects actual on-farm conditions, making it possible to utilize it in model training that can generalize to different farming conditions.

This dataset is designed to facilitate ongoing research for automatic fruit disease detection and classification and thus improve crop management practice. The dataset is an invaluable resource for developing AI-powered tools that will allow farmers to detect diseases at an early stage, optimize the utilization of resources, reduce dependence on chemicals, and optimize agricultural productivity and food safety in Bangladesh and other similar regions.

## 3 Data Description

In recent years, the use of image sets for plant disease detection has become more important, driven mostly by increased use of Artificial Intelligence in agri-technology. Although common datasets like PlantVillage helped researchers in making strides, most of them are only interested in diseases found on leaves and do not pay much attention to those manifested on fruits. This is particularly true for brinjal, a key vegetable crop in Bangladesh. Existing brinjal datasets are characterized primarily in terms of leaves and do not address adequately the variety of diseases that afflict the fruit. Single research groups have created small sets of images, but they're not published or disseminated to the wider scientific and farming communities. Such lack of brinjal fruit disease data is a significant roadblock to the development of useful detection tools. To fill this lacuna, we have developed a new, authentic dataset consisting entirely of images of brinjal fruit diseases captured directly from farms across Bangladesh. These images reflect real farm conditions, with diverse weather and environmental settings, which affect disease growth and appearance. Brinjal or eggplant is one of the four most important vegetable crops that are being grown in Bangladesh and that contribute to the livelihood of many small farmers. Fruit diseases reduce yields and cause economic losses, thus early and accurate diagnosis is crucial. Our data collection contains 1823 high-resolution images that are hand-tagged to five categories: Phomopsis Blight, Shoot and Fruit Borer, Fruit Cracking, Wet Rot, and Healthy Fruit. Our data collection is expected to spur research in plant pathology and assist in the construction of reliable AI models for early disease identification. Finally, we hope that this work will help improve crop management practices so that farmers can enhance the quality of their harvest and generate a better income.

During January and February 2025, a particular dataset based on brinjal fruit diseases was created by using images that were collected from major agricultural sites of Bogura, Dhaka. Images were taken under real field conditions, in various weather conditions—sunny morning, cloudy afternoon, and foggy morning—with temperature ranging from 18 °C to 24 °C and humidity ranging between 65 % and 80 %. High-resolution phones such as the Poco F3, Samsung A52 were used to obtain sharp and high-quality images. In contrast to most existing collections that are leaf-oriented infections, this dataset specifically targets fruit-oriented diseases. It has five clearly demarcated classes: Phomopsis Blight, which causes brown spots and internal rot due to Phomopsis vexans [1]; Fruit and Shoot Borer, an insect (Leucinodes orbonalis) that consumes fruit and stem from within [2]; Brinjal Fruit Cracking, a disorder typically linked to uneven moisture content or rapid growth, leading to cracked skin [3]; Brinjal Wet Rot, a fungal infection due to Choanephora cucurbitarum and Rhizopus Stolonifer, with soft, decaying tissue [4]; Healthy Brinjal, showing fruits with no visible disease. Every image of the dataset was checked carefully, annotated, and verified with the help of Daffodil International University's Department of Agriculture so that it is of the required standards to detect diseases accurately and can be used in machine learning applications.

**Fig. 1:**
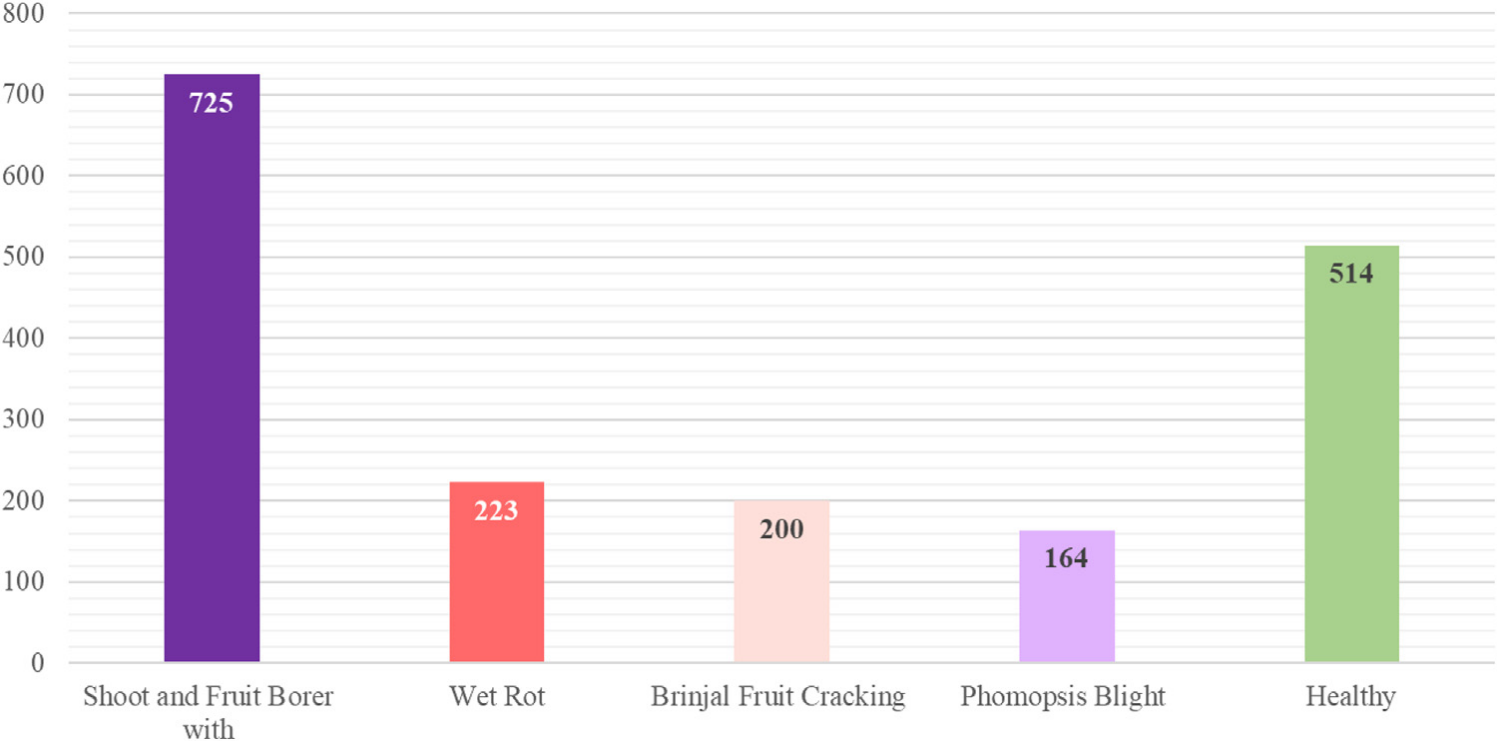
Dataset distribution graph.

Table 1 and Fig. 1 present the distribution of images over the different disease classes in our dataset, which is particularly targeting fruit-related diseases. A total of 2223 images was initially gathered from various sources; however, after scrupulously eliminating duplicates, biased samples, and poor-quality images inappropriate for training a model, the final dataset consists of 1823 good-quality images. These photos fall under the following categories: 200 Fruit Cracking, 514 Healthy Brinjal, 161 Phomopsis Blight, 725 Fruit and Shoot Borer, and 223 Wet Rot.

**Table 1:** Category-wise data distribution.

Category	Number of original images
Shoot and Fruit Borer	725
Healthy	514
Wet Rot	223
Brinjal Fruit Cracking	200
Phomopsis Blight	161
Total	1823

We can see from the Table 2 that the observations are divided into five classes representing an inconsistent brinjal fruit condition. Shoot and Fruit Borer (SFB) is a product of infestation by larvae of Leucinodes orbonalis, leading to internal fruit rot and decay. Wet Rot (WR), a product of infestation by Colletotrichum spp., forms water-soaked lesions that form, blacken, and rot. Brinjal Fruit Cracking (BFC) is a physiological disorder caused by environmental stress, leading to cracking of the fruit skin. Phomopsis Blight (PB), caused by infection of the pathogen Phomopsis vexans, leads to dark sunken lesions resulting in internal rotting. Brinjal fruits that are healthy are firm, smooth, and free from any defect, indicating good health and crop management. These classes help in the identification and knowledge of various diseases and conditions of brinjal fruit quality.

**Table 2:** Class-wise data description with visualization.

Class	Definition	Sample
Shoot and Fruit Borer	This disease is caused by the larval stage of the insect *Leucinodes orbonalis*. The larvae bore into both shoots and fruits, causing internal damage that is not immediately visible from the outside. Infested fruits show exit holes and often rot from the inside, making them unfit for consumption or sale. BFSB is one of the most destructive pests affecting brinjal production.	
Wet Rot	Caused by *Colletotrichum* spp., this disease presents as small, water-soaked lesions on the fruit that expand and turn dark with time. These lesions often crack or sink into the fruit, resulting in tissue breakdown and rot. The disease reduces the visual and structural quality of the fruit, affecting its commercial value.	
Brinjal Fruit Cracking	Fruit cracking in brinjal is a physiological disorder often caused by irregular watering, rapid fruit growth, or sudden changes in humidity. Cracks typically appear on the outer skin of mature fruits and may expose the inner flesh, making the fruit vulnerable to secondary infections and reducing market value. It is not caused by pathogens but is often aggravated by environmental stress and nutrient imbalance.	
Phomopsis Blight	Phomopsis fruit rot is caused by the fungal pathogen Phomopsis vexans. It typically manifests as dark, sunken lesions on the surface of the brinjal fruit, which gradually enlarge and lead to internal decay. The disease thrives in warm, humid environments and significantly reduces fruit quality and marketability due to soft rotting.	
Healthy	A healthy brinjal fruit is firm, smooth, and glossy with a uniform shape and deep color (typically purple, green, or white depending on the variety). It is free from visible deformities, lesions, or insect damage. Healthy fruits contribute to high marketability and reflect proper crop management and disease control practices in the field.	

We can see from the Table 3 that various studies have been accountable for creating leaf disease datasets for brinjal and other crops. The datasets, as referenced from [[5], [6], [7], [8], [9]], are focused on different aspects of leaf disease classification, such as large-scale datasets, balanced classes, and real-world, all with the goal of improving model training for leaf disease detection in real field environments. However, these datasets are primarily focused on diseases of leaves rather than fruits. Although a few datasets [10,11] have attempted to focus on brinjal (eggplant) fruit diseases, they remain highly limited in class diversity. Several important and economically significant diseases are either underrepresented or entirely absent, reducing the usefulness of these datasets for real-world disease detection. Moreover, to the best of our knowledge, no comprehensive research paper specifically addressing brinjal fruit disease dataset is currently available. This highlights a clear research gap and underscores the need for a more complete and publicly accessible dataset dedicated to brinjal fruit diseases.

**Table 3:** Comparison of existing works on brinjal.

References	Year	Disease Type	Class	Contribution
[5]	2025	Leaf Disease	6	Large-scale leaf disease dataset under natural field conditions
[6]	2024	Leaf Disease	11	Focus on classification with balanced leaf disease categories
[7]	2023	Leaf Disease	7	Leaf disease images for improved model training
[8]	2024	Leaf Disease	10	Comprehensive dataset supporting multi leaf disease detection
[9]	2025	Leaf Disease	6	Extensive image set focusing on real-world application scenarios
[10]	2022	Fruit Disease	4	Image Dataset on Brinjal Fruit Disease
[11]	2022	Fruit Disease	4	Image Dataset on Brinjal Fruit Disease
Our Dataset	2025	Fruit Disease	5	This is the first known collection to feature 1823 distinct and non-duplicate images of brinjal fruit diseases, which can support early detection of fruit infections.

On the other hand, our dataset developed in 2025 is the first one that is specifically known to be solely dedicated to brinjal fruit diseases with 5 classes. It comprises 1823 non-redundant and unique images representing five significant fruit diseases of brinjal. This dataset addresses a significant research gap of existing literature since it is the first one to aim for fruit-related disorders such as Phomopsis Blight, Fruit and Shoot Borer, Fruit Cracking, Wet Rot, and Healthy Brinjal. The novelty of our dataset lies in its one-pointed focus on fruit diseases, with different disease classes and the ability to support early detection of fruit infection, hence making it an invaluable resource towards enhancing both research and application in agricultural disease control. Although the dataset includes five major brinjal fruit diseases, several additional diseases commonly found in Asian growing conditions such as Alternaria Fruit Rot and Fusarium disorders were not visible during the data collection period. As a result, these categories could not be included.

## 4 Experimental Design, Materials and Methods

**Fig. 2:**
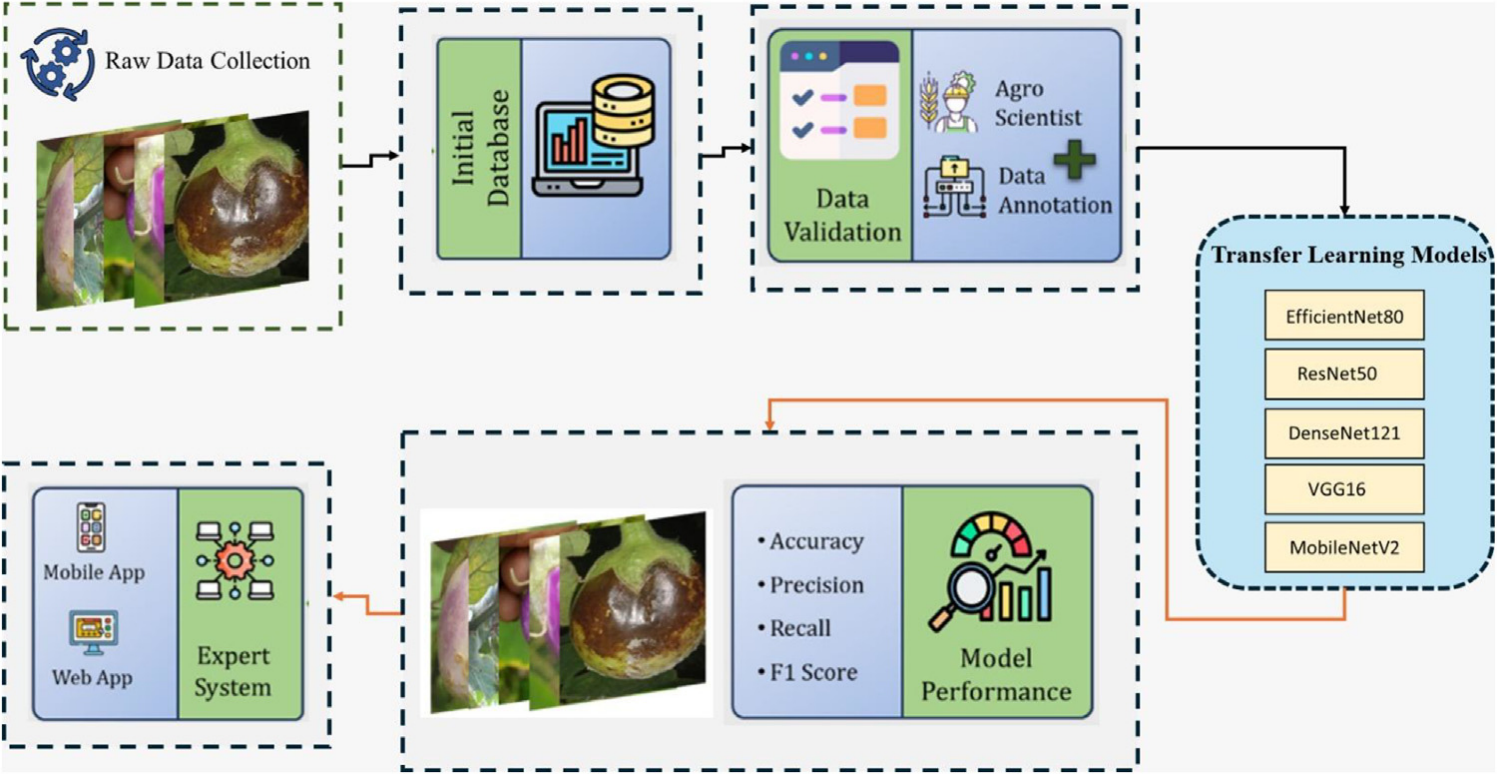
Step by step working procedure of fruits and its condition classification.

The research design for the brinjal dataset followed a structured approach, beginning with the capture of fruit images using two mobile cameras to introduce diversity in lighting, angles, and image quality. The dataset includes five main categories: Phomopsis Blight, Fruit and Shoot Borer, Brinjal Fruit Cracking, Wet Rot, and Healthy Brinjal. Agricultural experts validated the dataset to ensure the accuracy of classifications. The process of dataset preparation and classification, leading to integration into an expert system, is illustrated in Fig. 2.

### 4.1 Materials and equipment’s specification

The data were collected over a period of days, January to February 2025, by two individuals from two different regions of Bangladesh, Bogura and Dhaka. The data were taken under different weather conditions of sunny, foggy, and cloud weather with temperature ranging from 18 °C to 24 °C and humidity ranging from 65 % to 80 % shows in Table 4. The images were captured using two mobile camera gadgets, which are Poco F3 with 48MP and Samsung A52 with 64MP. Collection of data employed mixed use of the devices according to availability and weather. For example, on 10th Jan 2025, the pictures were taken with a mix of 60 % Poco F3 and 40 % Samsung A52 in Bogura, and on 3rd Feb 2025, all the pictures were taken using the Poco F3 (100 %) in Dhaka. This heterogeneity of strategy, combined with the heterogeneity of lighting and environmental conditions, ensures that the dataset captures a wide range of image qualities and viewpoints and is therefore more robust to train and test models.

**Table 4:** Details of camera specification.

Date	Weather	Time	Temp ( °C)	Humidity ( %)	Camera Device	Location
10 Jan 2025	Sunny	Morning	18	75	Poco F3 (60 %), Samsung A52 (40 %)	Bogura
15 Jan 2025	Foggy	Afternoon	20	80	Samsung A52 (100 %)	Dhaka
20 Jan 2025	Cloudy	Noon	22	70	Poco F3 (50 %), Samsung A52 (50 %)	Dhaka
23 Jan 2025	Sunny	Morning	19	78	Poco F3 (100 %)	Bogura
30 Jan 2025	Cloudy	Afternoon	21	72	Samsung A52 (70 %), Samsung A52 (30 %)	Dhaka
3 Feb 2025	Sunny	Noon	24	65	Poco F3 (100 %)	Dhaka
5 Feb 2025	Cloudy	Morning	23	68	Samsung A52 (100 %)	Bogura

### 4.2 Geographical location

The fruit image dataset was systematically collected through extensive field surveys conducted in Bangladesh between 10 January 2025 to February 5, 2025. The selection of collection sites was strategically planned to encompass diverse geographical, environmental, and agricultural conditions, ensuring a well-balanced and representative dataset. The geographic locations of the brinjal data collection sites are as follows:

• Bogura, Bangladesh, Latitude: 24.8483° N, Longitude: 89.2220° E, Zone: Rajshahi, Country: Bangladesh
• Dhaka, Bangladesh, Latitude: 23.8103° N, Longitude: 90.3200° E Zone: Dhaka, Country: Bangladesh

### 4.3 Data validation and annotation

The data validation and annotation process involved thorough inspection and verification by agricultural experts to ensure the accuracy of fruit classifications into five distinct categories: Healthy Brinjal, Phomopsis Blight, Fruit and Shoot Borer, Fruit Cracking, and Wet Rot. Expert agronomists carefully assessed the images to confirm the correct categorization of each fruit, considering various disease symptoms and fruit conditions. After capturing the images, experts conducted field inspections to verify the presence of specific disease traits. The validation process adhered to a systematic approach that guaranteed the dataset's reliability and accuracy for machine learning applications. Disease labels in this dataset were assigned based on field-level visual symptoms only, without laboratory or molecular confirmation. All visual diagnoses were cross-checked by Professor Dr. M. A. Rahim (Head, Department of Agricultural Science, DIU) to improve reliability, but the dataset should be considered visually identified rather than pathogen-verified.

### 4.4 Preprocessing

To preprocess the dataset, we employed duplicate image removal techniques (shows in Algorithms 1) to ensure the dataset had only unique, high-quality images. Since the dataset was captured by two individuals from different regions and under varying conditions, there was a possibility of duplicate images being captured. For this reason, we employed an image deduplication process using perceptual hashing. A similarity threshold of 0.90 was applied, and 73 duplicate images were eliminated. This process involved converting images into grayscale format, resizing them to the same size, and computing a perceptual hash value of every image. Hashing these values one against the other helped us identify and remove duplicate images and thus make sure that unique samples only were counted in the dataset. After the removal of duplicates, the remaining images were annotated thoroughly in accordance with visual verification and expert labelling. It maintained uniformity in the dataset by categorizing each of the images into one of the five classes of diseases: Healthy Brinjal, Phomopsis Blight, Fruit and Shoot Borer, Fruit Cracking, and Wet Rot. This preprocessing step was done to eliminate redundancy, improve dataset quality, and make the images used for model training diverse and reflective of real conditions.

**Algorithm 1:** Duplicate value remove algorithms.

**Algorithm 1** Image Deduplication Using Perceptual Hashing	

Input:	Input: Image collection ImgSet = {img₁, img₂, …, img_n_} containing n images
Output:	Filtered collection UniqueImgs containing only distinct images
Stage −1:	Initialize an empty set for storing hash values: HashRegistry ← {}
Stage −2:	Initialize an empty list for storing unique images: UniqueImgs ← {}
Stage −3:	**FOR each photo in ImgSet:**
Stage −4:	Convert photo to grayscale format
Stage −5:	Resize photo to a uniform size (height, width)
Stage −6:	Compute perceptual hash value hashVal using the pHash algorithm
Stage −7:	**IF hashVal is not in HashRegistry:**
Stage −8:	Add hashVal to HashRegistry
Stage −9:	Append photo to UniqueImgs
Stage −10:	**ELSE:**
Stage −11:	Label photo as a duplicate (do not add to output)
Stage −12:	**END IF**
Stage −13:	**End FOR**
Stage −14:	Return Unique Imgs as the deduplicated image set

### 4.5 Model implementation

We employed several machine learning models like ResNet50, DenseNet121, MobileNetV2, EfficientNetB0, and VGG16 to evaluate the performance of the brinjal fruit disease dataset. Preprocessing involved a series of steps that prepare the dataset to be used for model training, which included resizing the images into a uniform dimension, employing data augmentation techniques to enhance dataset diversity, and performing a train-test split to ensure it's being tested in the correct way. After model training, from Table 5, we observed the following results: MobileNetV2 achieved a peak accuracy of 93.98 %, followed by the remaining models, which all achieved high accuracies, with ResNet50 achieving 93.88 %, DenseNet121 achieving 93.78 %, and EfficientNetB0 achieving 93.89 %. Although the VGG16 model achieved slightly lower, it achieved an accuracy of 93.22 %. Precision, recall, and F1 measures were all strong across all the models and therefore confirm that they can be used to classify the various classes of fruit diseases—Healthy Brinjal, Phomopsis Blight, Fruit and Shoot Borer, Fruit Cracking, and Wet Rot. The results confirm that the dataset can be utilized for training deep learning models and therefore can be utilized to develop automated systems for detecting diseases in brinjal crops. The complete code, along with augmentation scripts and model development, is publicly available in our GitHub repository [12].

**Table 5:** Performance of applied deep learning models.

Model	Class	Accuracy	Precision	Recall	F1 score
ResNet50	Shoot and Fruit Borer (SFB)	0.9388	0.89	087	0.88
	Wet Rot (WR)		0.96	0.97	0.97
	Brinjal Fruit Cracking (BFC)		0.97	0.97	0.97
	Phomopsis Blight (PB)		0.94	0.94	0.94
	Healthy		0.94	0.94	0.94
DenseNet121	Shoot and Fruit Borer (SFB)	0.9378	0.85	0.89	0.87
	Wet Rot (WR)		0.97	0.99	0.98
	Brinjal Fruit Cracking (BFC)		0.96	0.97	0.96
	Phomopsis Blight (PB)		0.95	0.94	0.95
	Healthy		0.96	0.90	0.93
MobilNetV2	Shoot and Fruit Borer (SFB)	0.9398	0.90	0.88	0.89
	Wet Rot (WR)		0.96	0.99	0.98
	Brinjal Fruit Cracking (BFC)		0.95	0.99	0.97
	Phomopsis Blight (PB)		0.95	0.92	0.94
	Healthy		0.96	0.94	0.95
EfficientNetB0	Shoot and Fruit Borer (SFB)	0.9389	0.90	0.88	0.89
	Wet Rot (WR)		0.96	0.99	0.98
	Brinjal Fruit Cracking (BFC)		0.95	0.99	0.97
	Phomopsis Blight (PB)		0.95	0.92	0.94
	Healthy		0.96	0.94	0.95
VGG16	Shoot and Fruit Borer (SFB)	0.9322	0.85	0.85	0.85
	Wet Rot (WR)		1.00	0.97	0.98
	Brinjal Fruit Cracking (BFC)		0.95	0.97	0.96
	Phomopsis Blight (PB)		0.90	0.98	0.94
	Healthy		0.96	0.89	0.92

Aside from its value for research, this information also opens huge opportunities for real-world applications, particularly in tracking supply chains and ensuring food security. In cold-chain supply, it can be used on installed IoT systems, including sensors and cameras with machine learning software in a bid to enable real-time fruit classification. These systems can be employed to monitor vital environmental parameters, such as temperature, humidity, and gas concentration, across fruit transportation and storage so that spoilage, contamination, or formalin adulteration can be detected early. Formalin-mixed fruit samples included in the dataset provide added value to ensure it can be utilized in regulatory inspections for curbing chemical adulteration. Additionally, the data set can be applied in smart inspection systems to guarantee quality, reduce food waste, and enable cloud-based systems to notify stakeholders of any quality deviations. To the consumer, the data set enables the development of mobile applications for assessing fruit quality via smartphone cameras, promoting responsible purchasing and public health awareness, particularly where food adulteration is high. Thus, the dataset not only aids in improving food quality and food safety but also plays a key role in supply chain transparency that works for farmers and consumers.

## Limitations

The images were gathered from a few selected regions in Bangladesh, which may reduce the generalizability of the dataset when applied to different environmental or agricultural settings. A noticeable imbalance in the number of images across disease classes is present, particularly for less common diseases like Rhizopus and Wet Rot. With over 700 images of Shoot and Fruit Borer compared to only 161 images of Phomopsis Blight. This imbalance may affect model fairness and benchmarking reliability. To address this, researchers may apply class-balancing techniques such as SMOTE, ADASYN, or targeted data augmentation where an augmentation code is available in our code repository [12]. Another limitation is that only visible disease symptoms were documented. Internal or early-stage signs that are not externally apparent were not captured, which may reduce the model’s usefulness for early detection. Moreover, the absence of temporal data restricts the ability to analyze fruit spoilage progression over time, which could be valuable for time-series-based models in food quality monitoring*.*

## Ethics Statement

We confirm that our study followed all ethical guidelines. No plants, animals, or people were harmed during the research. Also, we did not collect any data from social media. All authors agree to follow the ethical rules needed for publishing in Data in Brief.

## CRediT Author Statement

**Md. Zahid Hasan:** Data Collection, Data Curation, Conceptualization, Writing – Review & Editing; **Abu Kowshir Bitto:** Data Collection, Data Curation, Conceptualization, Methodology, Visualization, Code Development, Writing; **Md. Hasan Imam Bijoy:** Data Collection, Data Curation, Methodology, Visualization, Validation, Writing; **Khalid Been Badruzzaman Biplob:** Data Curation, Code Development; **Mohammad Mahadi Hassan:** Funding*,* Writing – Review & Editing; **Mohammad Shohel Rana:** Funding*,* Writing – Review & Editing; **Abdul Kadar Muhammad Masum:** Funding*,* Writing – Review & Editing.

## Data Availability

Mendeley DataBrinjalFruitX: A Field-Collected Image Dataset for Machine Learning and Deep Learning-Based Disease Identification in Brinjal Fruits (Original data).
